# Transferred by exosomes-derived MiR-19b-3p targets PTEN to regulate esophageal cancer cell apoptosis, migration and invasion

**DOI:** 10.1042/BSR20201858

**Published:** 2020-11-23

**Authors:** Qingxin Zeng, Ziyi Zhu, Lijiang Song, Zhengfu He

**Affiliations:** Department of Thoracic Surgery, Sir Run Run Shaw Hospital, Zhejiang University School of Medicine, China

**Keywords:** esophageal cancer, exosomes, migration, miR-19b-3p, PTEN pathway

## Abstract

The present study aims to investigate the relationship between miR-19b-3p and esophageal cancer (ESCA), and to detect the effects of miR-19b-3p transferred by exosomes on the phenotype of EC9706 cells. The expression of miR-19b-3p was detected by starBase analysis and real-time quantitative PCR (RT-qPCR). The target genes of miR-19b-3p were predicted by TargetScan and further verified by luciferase analysis. The mRNA and protein expression levels of PTEN and EMT-related genes were detected by RT-qPCR and Western blotting. The effects of miR-19b-3p transferred by exosomes and its target genes on the apoptosis, migration and invasion of EC9706 cells were studied by establishing a co-culture model of donor cells. The expression of miR-19b-3p in ESCA plasma, cells and exosomes was significantly up-regulated. miR-19b-3p transferred by exosomes could significantly reduce EC9706 cells apoptosis rate, promote cell migration and invasion, and could target the inhibition of PTEN expression. PTEN overexpression promoted apoptosis, inhibited cell migration and invasion, down-regulated the expression of MMP-2 and vimentin, and up-regulated E-cadherin expression; however, these effects could be partially reversed by miR-19b-3p. In summary, our results reveal that miR-19b-3p transferred by exosomes can target PTEN to regulate ESCA biological functions in the receptor EC9706 cells.

## Introduction

Esophageal cancer (ESCA) is a malignant tumor that seriously threatens human health. With the advancement of technology and the development of drug research, the diagnosis and treatment of ESCA have made certain progress [1]. Among them, surgical resection is the main treatment method of ESCA; however, cancer cell metastasis could still occurred to patients and eventually leads to their deaths after treatment [2]. Tumor metastasis is the leading cause of ESCA treatment failure and patient death [3]. So far, the specific mechanism of ESCA invasion and transfer remains enigmatic [4]. Among them, whether the transmission of information between highly heterogeneous tumor cells in tumor tissue has an effect on tumor metastasis is a frontier scientific question to be addressed [5]. Thus, studying tumor microenvironment may provide a bran-new way for the diagnosis and treatment of tumor.

Studies have shown that tumor cells can interact with the microenvironment to promote tumor growth [5,6]. Exosomes play an important effect in information exchange between tumor and microenvironment [7]. Exosomes, small bilayer-membrane-encapsulated vesicles released by cells under physiological and pathological conditions [8], can affect tumor immunity, tumor invasion and metastasis, angiogenesis and so on through different molecular mechanisms [8]. It has been reported that MT1-MMP released by exosomes secreted by melanoma and fibrosarcoma can cause collagen degradation and promote tumor invasion and metastasis [9]. Liao et al. found that miR-21 in ESCA exosomes was taken up by recipient cells to target PDCD4 and activate the downstream c-JNK signaling pathway to promote the invasion and migration of recipient cells [10]. However, there are still few reports on ESCA exosomes.

Tumor cells release a variety of exosomes containing miRNA, and reach the metastatic site before the cancer cells, stimulating the metastasis of the tumor microenvironment, thereby promoting tumor metastasis [11]. For example, studies showed that exosomal-transferred miR-93-5p promotes proliferation of recipient ESCA cells [12]. Exosome-mediated delivery of functionally active miRNA-142-3p inhibitor reduces tumorigenicity of breast cancer *in vitro* and *in vivo* [13]. Increasing evidence demonstrated that miRNAs are involved in tumor progression. MiR-19b-3p promotes human pancreatic cancer Capan-2 cells proliferation by targeting phosphatase and tension homolog (PTEN) [14]. MiR-19b-3p is associated with the risk and severity of knee osteoarthritis [15] and induced extracellular matrix degradation and inflammatory injury in chondrocytes [16], and can be used as a potential biomarker for diseases such as Alzheimer’s disease and gastric cancer [17,18]. Interestingly, miR-19a is significantly overexpressed in the plasma of ESCA patients, and ESCA can also be used as a potential biomarker for poor prognosis [19]. In addition, some scholars found that miR-19b-3p inhibits breast cancer cell proliferation by regulating PI3K/Akt pathway [20]. In addition, exosomal miR-19b-3p of tubular epithelial cells promotes M1 macrophage activation in kidney injury [21]. Study have reported that cancer stem cells exosomes transported miR-19b-3p into clear cell renal cell carcinoma cells and initiated EMT promoting metastasis [22]. However, the molecular regulatory mechanism of exosome-transferred miR-19b-3p in ESCA is unknown. Given that, the study explored the relationship between exosome-transferred miR-19b-3p and the pathogenesis of ESCA, and the roles of exosome-transferred miR-19b-3p on the apoptosis, migration, and invasion of EC9706 cells.

## Materials and methods

### Collection of blood samples

Peripheral blood (8–10 ml) was collected in an EDTA anticoagulation tube before operation, and gently mixed at 4°C, 1900 × ***g***, and the peripheral blood sample was centrifuged for 10 min. The supernatant was carefully taken as plasma. At 4°C, 3000 × ***g***, the obtained plasma was centrifuged again for 15 min, and the plasma was aspirated carefully without touching the bottom and side sediments. Plasma samples were stored in a refrigerator at −80°C for later use.

### Cell, culture and transfection

Human esophageal cancer cell lines (EC9706, C0302, https://sgdbio.chemdrug.com/, CA) were used in the present study. FBS (30067334, ThermoFisher, U.S.A.) was centrifuged at 200,000 × ***g*** for 6 h to remove exosomes. About 10% exosomes-free FBS and 1% penicillin/streptomycin (15140163, ThermoFisher, U.S.A.) were added to RPMI-1640 medium (61870044, ThermoFisher, U.S.A.) to get a cell complete medium. The EC9706 cells were quickly revived in a 37°C water bath, and then cultured in a 37°C, 5% CO_2_ incubator with fresh medium. After reaching 80–90% confluence, the cells were digested with 0.25% trypsin and were then passaged.

The pcDNA3.1 (V79520) and PTEN (A15629, A15630) were purchased from ThermoFisher Company, and mimic control (miR1N0000001-1-5), miR-19b-3p mimic (MQPS0000777-1-100) were obtained from RIBOBIO Company (https://www.ribobio.com/). EC9706 cells were transfected with miR-19b-3p mimic or PTEN. Cell transfection was performed according to the manufacturer’s protocol (Lipofectamine 3000, L3000015, ThermoFisher). After the cells were transfected for 36 h at 37°C, EC9706 cells (1 × 10^5^ cells/well) transfected with miR-19b-3p mimic or PTEN were used as donor cells and seeded into the upper chamber of Transwell culture plates (0.4 μm), ordinary EC9706 cells (1 × 10^5^ cells/well) were seeded into the lower chamber as recipient cells and co-cultured for 24 h. Finally, the transfected cells were analyzed.

### Immunofluorescence

Transient cell transfection: EC9706 cells (donor) transiently transfected with cy3-labeled (red) miR-19b-3p were co-cultured with GFP labeled (green) recipient EC9706 cells. EC9706 cells (1 × 10^5^ cells/well) transfected with cy3-miR-16-5 mimic as donor cells were seeded into the upper chamber of Transwell culture plates (0.4 μm), and GFP-EC9706 cells (1 × 10^5^ cells/well) as recipient cells. After co-culture for 24 h, the contents of cy3-miR-19b-3p in recipient cells were observed using a fluorescence microscope.

### Exosomes isolation

Exosomes were isolated as previously mentioned [12]. In short, the extractions of extracellular bodies were performed by differential centrifugation (300 × ***g***, 10 min; 1200 × ***g***, 20 min; and 10,000 × ***g***, 30 min; 4°C) and ultracentrifugation (200,000 × ***g***, 2 h; 100,000 × ***g***, 2 h; 4°C). After the supernatant was discarded, the exosomes were suspended using PBS (100 μl) and then stored at −80°C. The exosomes were observed and photographed with a transmission electron microscope. Western blot was used to detect the expression of exosome surface marker molecule CD63 (ab134045, Abcam, Cambridge, MA, U.S.A.).

### Real-time quantitative PCR

The mRNA expression of miR-19b-3p, PTEN, MMP-2, E-cadherin and vimentin were detected by real-time quantitative PCR [23]. Samples such as cells or exosomes were lysed for 5 min at room temperature in 1 ml of Trizol, 200 μl of chloroform was then added to the mixture and quickly and vigorously mixed for 15 s. Then the mixture could stand and centrifuged (4°C, 12,000 × ***g***, 15 min). The supernatant was added with 500 μl of isopropanol, left at room temperature for 10 min, and centrifuged again for 15 min. The supernatant was discarded, 1 ml of 75% ethanol was added, and the mixture was centrifuged for 5 min. The supernatant was discarded and dried, and the RNA solution was obtained by dissolving with an appropriate amount of RNA-free water. The reverse transcription of cDNA was used by PrimeScrip II 1st Strand cDNA Synthesis Kit (6210B, Takara, Japan). The Verso 1-step RT-qPCR Kit (A15300; Thermo Scientific, U.S.A.) and opticon RT-PCR ABI 7500 detection system (Life Technology, U.S.A.) were used. The relative expression levels were calculated with the 2^−ΔΔCt^ method [24]. U6 and GAPDH served as housekeeping genes. The primers sequences were as listed in Table 1.

**Table 1:** Primers used for reverse transcription-quantitative PCR

Gene	Primer sequence (5′-3′)
miR-19b-3p	F: ACTGAGTCGTATCCAGTGCAA
	R: GTATCCAGTGCGTGTCGTGG
U6	F: CTCGCTTCGGCAGCACA
	R: AACGCTTCACGAATTTGCGT
PTEN	F: TGGATTCGACTTAGACTTGACCT
	R: GGTGGGTTATGGTCTTCAAAAGG
MMP-2	F: TACAGGATCATTGGCTACACACC
	R: GGTCACATCGCTCCAGACT
E-cadherin	F: CGAGAGCTACACGTTCACGG
	R: GGGTGTCGAGGGAAAAATAGG
Vimentin	F: GACGCCATCAACACCGAGTT
	R: CTTTGTCGTTGGTTAGCTGGT
GAPDH	F: GGAGCGAGATCCCTCCAAAAT
	R: GGCTGTTGTCATACTTCTCATGG

### Western blotting analysis

The protein expression of PTEN, MMP-2, E-cadherin, and vimentin were detected by Western blot [25]. The extraction of total protein was used by RIPAlysis buffer (89901, Thermo Scientific, U.S.A.) containing PMSF protease inhibitor [36978]. And determination of protein concentration was used with Protein Assay Kit (A53227). The obtained protein sample (20 μg) was mixed with the loading buffer, and then placed in a water bath in a 95-water bath for 5 min. A 10% SDS-polyacrylamide gel (SDS-PAGE) was prepared, and the protein was electrophoresed to 80 V constant pressure until separation. At the interface between the gel and the concentrated gel, the voltage was changed to 120 V, and then the protein was transferred to the PVDF membrane (HVLP04700, Millipore, U.S.A.) under a constant current of 200 mA for 2 h. After blocking for 2 h, primary antibodies as PTEN (1/1000, #9552, 54 KD, Cell Signaling Technology, U.S.A.), MMP-2 (1 μg/ml, ab37150, 72 KD, Abcam), E-Cadherin (1/1000, #14472, 80KD, CST), Vimentin (1/1000, ab92547, 54 KD, Abcam), and GAPDH (1/5000, ab8245, 36KD, Abcam) were incubated at 4°C overnight. Corresponding secondary antibodies such as Goat Anti-Rabbit IgG H&L (HRP) (1:5000, ab205718) and Goat Anti-Mouse IgG H&L (HRP) (1:5000, ab205719) were incubated at room temperature for 2 h, and TBST was used to wash the membrane for three times, 10 min/time. After developing color with a chemiluminescence agent [SignalFire ECL reagent (#6883, CST)], imageJ (version 5.0, Bio-Rad, U.S.A.) analyzed the relative content of the target protein in the sample.

### Flow cytometry

The apoptosis rates of donor-derived exosome miR-19b-5p plasmids to EC9706 cells were determined by flow cytometry. The kit was purchased from Beyotime (C1062S, CA). The simple steps are as follows: Annexin V-FITC (5 μl) and PI staining solution (10 μl) were added to the EC9706 cell suspension (1 × 10^5^/wells); afterward, the cells were incubated at room temperature in the dark for 10–20 min and were then placed in an ice bath. Apoptosis rate was detected with flow cytometry (342973, BD Biosciences, U.S.A.).

### Wound healing assay

Donor cells and recipient cells were co-cultured in Transwell culture plates at a density of 1 × 10^5^/well for 24 h. We marked a “1” on the cells with a sterile pipette tip, then added fresh medium to incubate the cells for 24 h and then observed and photographed the wound healing with a microscope (BZ-8100, Keyence, Japan) and Image-pro Plus 4.1 analysis software (Media Cybernetics Company, U.S.A.).

### Transwell

Invasion rates of EC9706 cells were detected by performing 24-well transwell (CLS3398, Sigma, Germany). After co-culturing the donor and recipient cells for 24 h, the recipient cells (1 × 10^5^ /well) were seeded into the Matrigel-lined (354230, BD, U.S.A.) upper chamber, whereas 600 μl of 20% FBS-containing medium was filled into the lower chamber. After 24 h of incubation, the Transwell chamber was removed from the incubator and washed with PBS. Subsequently, the upper matrigel and cells were wiped off with a cotton swab. Membranes were then fixed with 95% ethanol for 10 min, stained with Crystal Violet for 10 min, washed with PBS three times, and observed under the microscope and photographed.

### Dual luciferase activity assay

The potential binding sites of miR-19b-3p and PTEN were predicted by TargetScan7.2. The pmirGLO (E1330; Promega, CA, U.S.A.) was used to establish PTEN-3′-UTR, and PTEN-3′-UTR mut luciferase Vectors. The liposomes were used to co-transfect miR-19b-3p mimic and recombinant reporter vector into the cells, and the transfected cells were cultured in a carbon dioxide incubator. Transfected cells were collected and detected with a Dual Luciferase Reporter Detection Kit (FR201-01, TransGen Biotech, CA), and the firefly luciferin signal and Renilla luciferin signal of the cells were determined with a luminescence meter (SpectraMaxL, Molecular Devices, U.S.A.).

### Statistical analysis

The data were expressed as mean ± standard deviation (SD), performed with Tukey’s post hoc test and one-way analysis of variance (ANOVA). The data were processed using SPSS 19.0 statistical software (Chicago, IL, U.S.A.). *P*<0.05 was considered as statistically significant.

## Results

### miR-19b-3p was highly expressed in ESCA patients

**Figure 1:**
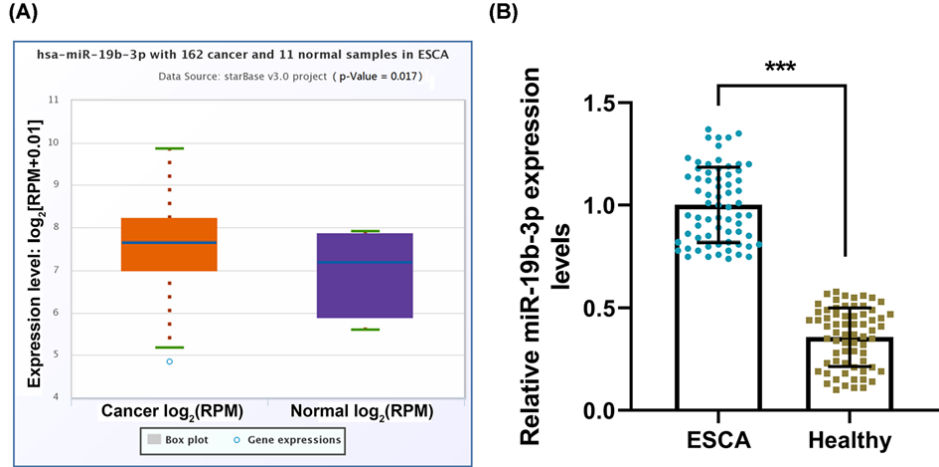
miR-19b-3p was highly expressed in esophageal cancer (**A**) StarBase v2.0 was used to analyze the expression of miR-19b-3p in the TCGA-ESCA database of esophageal cancer (including 162 cancer samples and 11 healthy samples). (**B**) RT-qPCR was used to compare the expression of miR-19b-3p in the plasma of healthy controls and patients with esophageal cancer (*n*=67). U6 was used as the internal reference gene of miRNA. Results were expressed as the mean ± SD. RT-qPCR: reverse transcription real-time quantitative polymerase chain reaction; ESCA: esophageal cancer; ***P<0.001 vs. ESCA; ESCA, esophageal carcinoma.

The TCGA-ESCA database was used to study the effect of miR-19b-3p on ESCA. Starbase analysis results showed that the miR-19b-3p expression in ESCA samples was significantly higher than normal samples (*P*<0.05; Figure 1A). To verify the database prediction results, RT-qPCR was used to compare the miR-19b-3p expression in the plasma of healthy controls and ESCA patients, showing that the expression of miR-19b-3p in the plasma of ESCA patients was apparently higher than that of healthy samples (*P*<0.001, Figure 1B).

### miR-19b-3p was transmitted between EC9706 cells via exosomes

**Figure 2:**
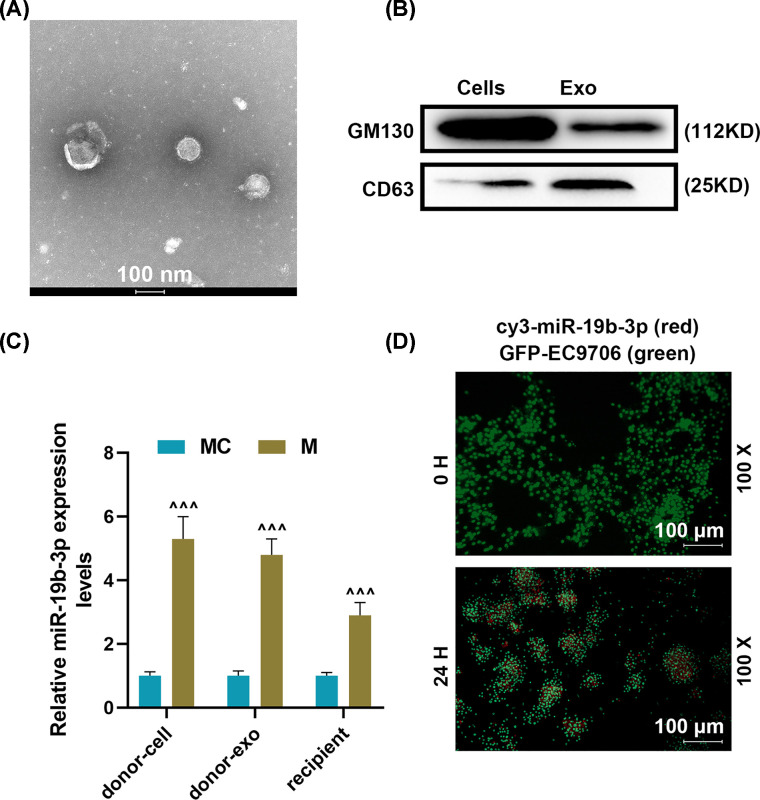
Effects of miR-19b-3p derived from exosomes on recipient human esophageal cancer cells (EC9706) (**A**) Identification of exosomes by transmission electron microscope. (**B**) Western blot was used to detect surface markers of exosomes. (**C**) RT-qPCR was used to detect miR-19b-3p expression in mimic control (donor cell), miR-19b-3p (donor cell), mimic control (donor exo), miR-19b-3p (donor exo), Mimic control (recipient), miR-19b-3p (recipient) group. (**D**) Fluorescence microscope was used to observe the transmission of cy3-miR-19b-3p between EC9706 cells through exosomes. U6 was used as the internal reference gene of miRNA. Results were expressed as the mean ± SD; RT-qPCR, reverse transcription real-time quantitative polymerase chain reaction; ^∧∧∧^*P*<0.001 vs. MC; MC, mimic control, M, miR-19b-3p mimic.

To study the role of miR-19b-3p transferred by exosomes on EC9706 cells, we isolated and identified exosomes. The results indicated that the exosomal marker CD63 was markedly overexpressed in exosomes rather than in cells. However, the expression of GM130 was highly expressed in cells but was hardly expressed in exosomes (Figure 2A,B). After miR-19b-3p transfection, miR-19b-3p expression was apparently up-regulated in donor cells and exosomes, while miR-19b-3p expression was also remarkable up-regulated in recipient cells (*P*<0.001, Figure 2C). After co-culture of EC9706 cells (donor cells) transfected with cy3- miR-19b-3p mimic and GFP-EC9706 cells (recipient cells) for 24 h, the cy3-miR-19b-3p content in the recipient cells was increased significantly (magnification × 100, scale bar = 100 μm, Figure 2D).

### miR-19b-3p transferred by exosomes reduced the apoptosis rates of the recipient EC9706 cells and promoted cell migration and invasion

**Figure 3:**
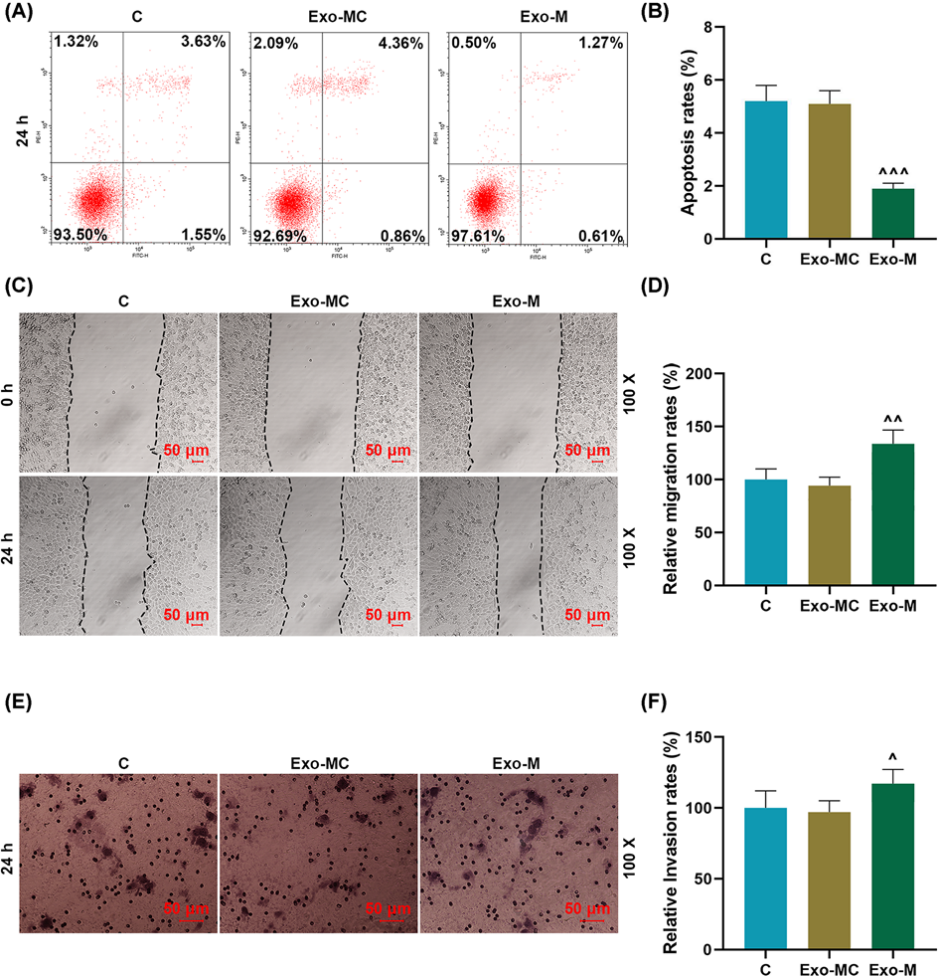
Effects of miR-19b-3p transferred by exosomes on the apoptosis rate, migration and invasion ability of EC9706 cells (**A** and **B**) Flow cytometry was used to detect the effects of exosomes miR-19b-3p on the apoptosis rate of EC9706 cells. (**C** and **D**) Wound healing assay was used to detect the effects of miR-19b-3p transferred by exosomes on the migration capacity of EC9706 cells. (**E** and** F**) Transwell assay was used to detect the effect of miR-19b-3p transferred by exosomes on the invasion of EC9706 cells; Exo, exosomes; MC, mimic control, M, mimic; ^∧^*P*<0.05, ^∧∧^*P*<0.01, ^∧∧∧^*P*<0.001 vs. Exo-MC.

The biological role of miR-19b-3p transferred by exosomes in the recipient EC9706 cells was further studied. Flow cytometry results showed that miR-19b-3p transferred by exosomes could significantly reduce the apoptosis rates of recipient cells (*P*<0.001, Figure 3A,B). Moreover, the migration rate in the Exo-M group was remarkable higher than that in the Exo-MC and Control groups (*P*<0.01, Figure 3C,D). In addition, the effect of donor-derived exosomes miR-19b-3p on the invasion of recipient EC9706 cells was examined. Compared with the control group, the invasion rates of the receptor EC9706 cells had no significant change in Exo-MC, but were significantly increased in the Exo-M group (*P*<0.05, Figure 3E,F).

### miR-19b-3p transferred by exosomes targeted PTEN expression in EC9706 cells

**Figure 4:**
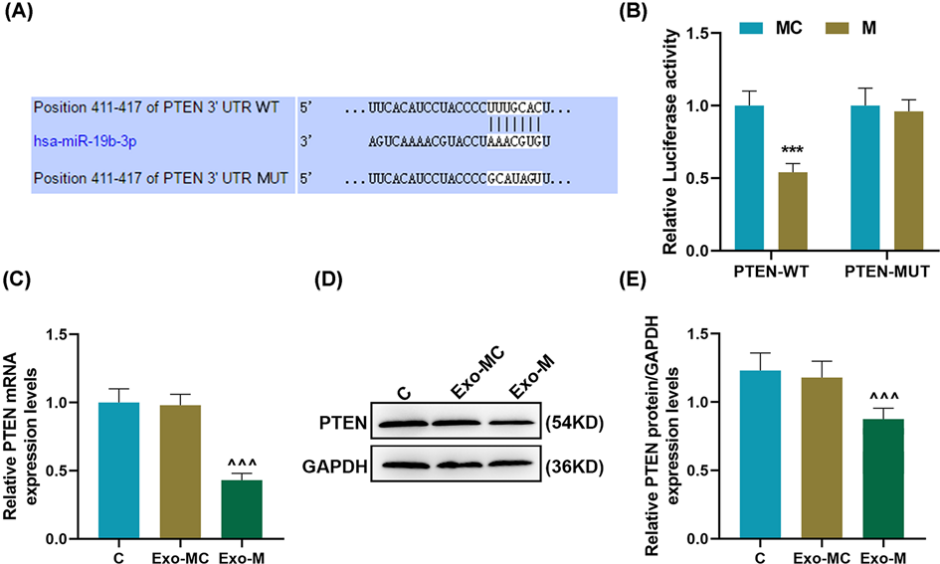
miR-19b-3p transferred by exosomes targeted the inhibition of PTEN expression in EC9706 cells (**A**) TargetScan was used to predict the targeted binding site of miR-19b-3p to PTEN. (**B**) The targeting relationship of miR-19b-3p and PTEN was verified by dual luciferase assay. (**C**–**E**) The effect of donor-derived exosomes miR-19b-3p on the expression of PTEN in recipient EC9706 cells was detected by RT-qPCR and Western blot. GAPDH was used as the internal reference gene of mRNA; RT-qPCR, reverse transcription real-time quantitative polymerase chain reaction; Exo, exosomes; MC, mimic control; ***P<0.001 vs. MC; ^∧∧∧^*P*<0.001 vs. Exo-MC.

In order to investigate the molecular mechanism of miR-19b-3p transferred by exosomes in EC9706 cells, TargetScan was used to predict the target genes of miR-19b-3p, demonstrating that the mRNA of PTEN contained miR-19b-3p targeting sites (Figure 4A). Luciferase analysis showed that miR-19b-3p up-regulation could significantly inhibit the fluorescence of PTEN-WT, but had no effect on the fluorescence of PTEN-MUT (*P*<0.001, Figure 4B). RT-qPCR and Western blot were used to detect the effect of donor-derived exosomes miR-19b-3p on the expression of PTEN in EC9706 cells after co-culture for 24 h, and found that miR-19b-3p transferred by exosomes could effectively target the inhibition of PTEN expression in recipient cells (*P*<0.001, Figure 4C–E).

### Overexpression of PTEN targeted by exosomes-transferred miR-19b-3p to regulate receptor EC9706 cell motility

**Figure 5:**
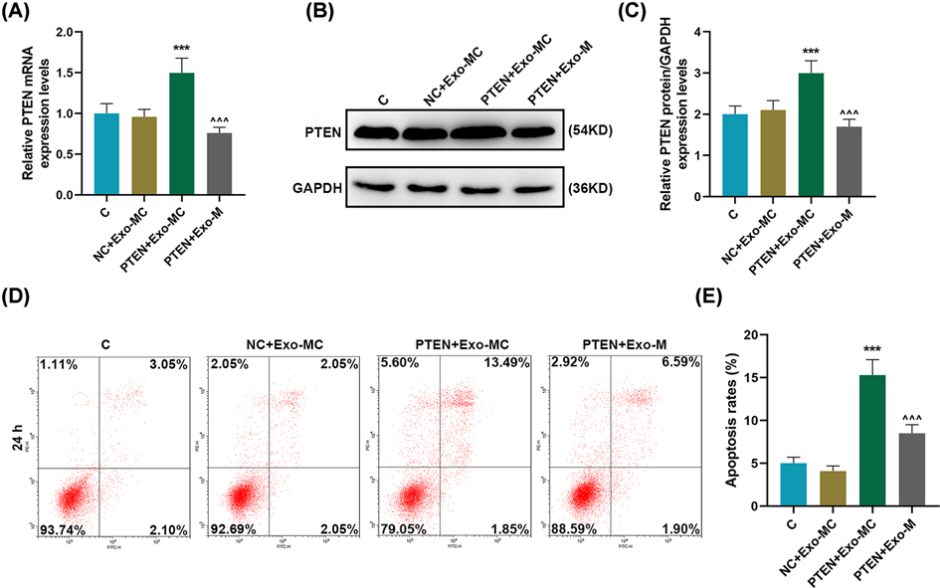
The effect of overexpression of PTEN on the apoptosis of EC9706 cell (**A–C**) RT-qPCR and Western blot were used to detect changes in PTEN expression in the Control, NC + Exo-MC, PTEN + Exo-MC, and PTEN + Exo-M groups. (**D** and** E**) Flow cytometry was used to detect the change of apoptotic rate in each group. GAPDH was used as the internal reference gene of mRNA. Results were expressed as the mean ± SD. RT-qPCR: reverse transcription real time quantitative polymerase chain reaction; Exo: exosomes; MC: mimic control, M: mimic; ***P<0.001 vs. NC + Exo-MC; ^∧∧∧^*P*<0.001 vs. PTEN + Exo-MC.

**Figure 6:**
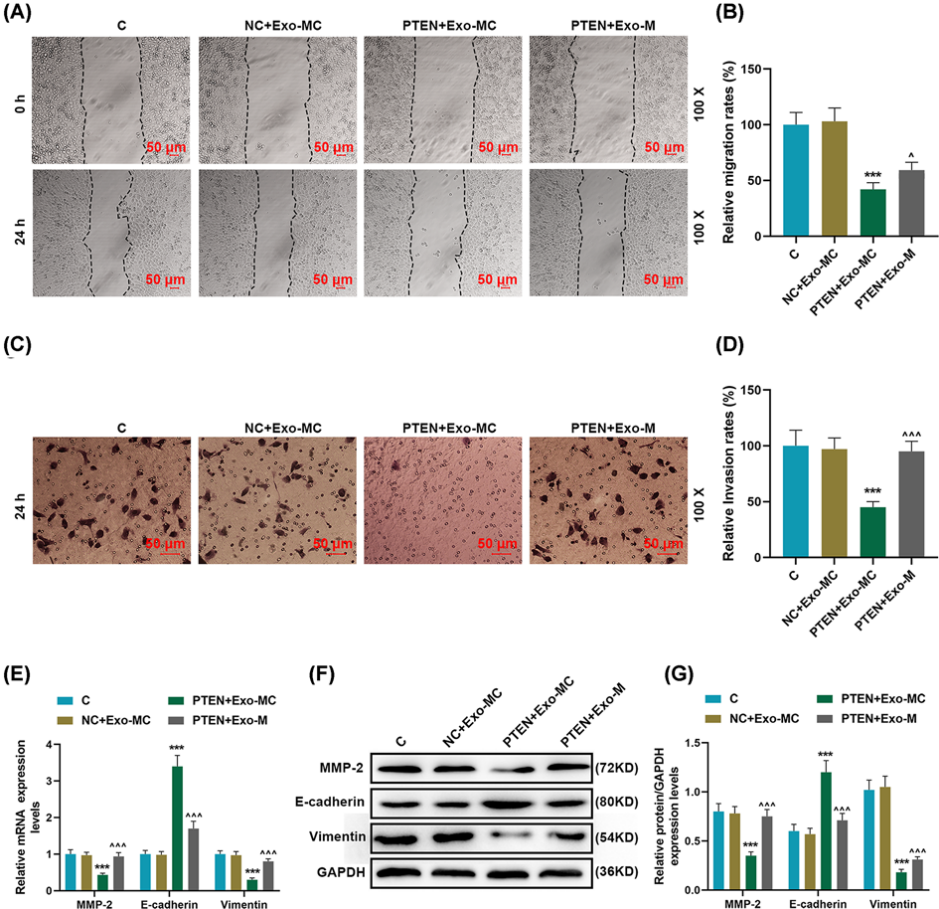
Overexpression of PTEN targeted by exosomes-transferred miR-19b-3p regulated the migration, invasion and EMT related proteins of EC9706 cells (**A** and** B**) Wound healing assay was used to detect the migration of receptor EC9706 cells in the Control, NC + Exo-MCl, PTEN + Exo-MC, PTEN + Exo-M groups. (**C** and **D**) Transwell assay was used to detect the invasive ability of EC9706 cells in each group. (**E**–**G**) RT-qPCR and Western blot were used to detect the expression of E-cadherin, vimentin, and MMP-2 in each group. GAPDH was used as the internal reference gene. Results were expressed as the mean ± SD. RT-qPCR: reverse transcription real-time quantitative polymerase chain reaction; Exo: exosomes; MC: mimic control, M: mimic; ***P<0.001 vs. NC + Exo-MC; ^∧^*P*<0.05, ^∧∧∧^*P*<0.001 vs. PTEN + Exo-MC.

In order to further understand the effects of exosomes-transferred miR-19b-3p and PTEN on regulating the motor capacity of the receptor EC9706 cells, overexpressing PTEN and miR-19b-3p transferred by exosomes were successfully transfected into cells for research. Our results showed that overexpression of PTEN could effectively promote the expression of PTEN in recipient cells, and miR-19b-3p transferred by exosomes could effectively reverse the up-regulation of PTEN expression (*P*<0.001, Figure 5A–C). PTEN overexpression could promote apoptosis, while miR-19b-3p transferred by exosomes significantly reversed the effect of PTEN overexpression on the apoptosis of recipient cells (*P*<0.001, Figure 5D,E). Compared with the NC + Exo-MC group, the migration rate in the PTEN + Exo-M group was apparently reduced, and the migration rate in the PTEN + Exo-M group was significantly higher as compared with that in the PTEN + Exo-MC group (*P*<0.05, Figure 6A,B). PTEN overexpression can significantly inhibit cell invasion, while miR-19b-3p transferred by exosomes significantly reversed the inhibitory effect of PTEN overexpression on cell invasion (*P*<0.001, Figure 6C,D). In addition, overexpression of PTEN could significantly down-regulate MMP-2 and vimentin, and up-regulate E-cadherin; and miR-19b-3p transferred by exosomes significantly inhibited the regulation of PTEN overexpression on EMT-related proteins (*P*<0.001, Figure 6E–G).

## Discussion

With in-depth study of the etiology of ESCA, ESCA is currently considered to be a complex disease with multiple factors, multiple genes and multiple stages of development [1,2]. As the molecular mechanism of ESCA development is still not fully elucidated, reliable and accurate molecular markers are still lacking for its early detection and diagnosis [4]. Therefore, exploring the related molecular mechanisms of ESCA metastasis and recurrence, and finding new tumor markers have become the focus and difficulty of ESCA research.

Exosomal research is a very popular field of biological research in recent years [26]. More and more *in vivo* and *in vitro* studies have found that exosomes can affect a variety of physiological and pathological processes [27]. Metastasis is the main challenge in the treatment of cancer and the main cause of cancer death, some researchers have reported that the interaction between exosomes and tumor *in situ* ultimately promotes tumor metastasis potential [27,28]. We isolated the exosomes of EC9706 cells by ultracentrifugation. Electron microscopy results and Western blotting experiments showed that exosomes were successfully isolated. With the discussion of the source of extracellular miRNA, more and more studies believe that exosomes may be an important carrier of extracellular miRNA [29].

miRNA plays an important regulatory role in ESCA and may become a new target for ESCA treatment [30]. Xie et al. compared the saliva supernatants of 32 ESCA patients and 16 healthy people and found that the miR-21 expression was significantly increased in the saliva supernatants of ESCA patients, providing certain reference value for ESCA diagnosis [31]. Meanwhile, miRNAs up-regulated in ESCA tissues include miR-16 [32], miR-25 [33], miR-155 and so on [34]. miR-19b-3p is abnormally expressed in many cancers such as breast cancer and pancreatic cancer [14,18,20]. For example, Huang et al. found that miR-19b-3p is up-regulated in nasopharyngeal carcinoma, and miR-19b-3p increases the radiation resistance of nasopharyngeal carcinoma cells by activating the TNFAIP3/NF-κB signaling pathway [35]. MiR-19b and miR-20a suppress apoptosis, promote proliferation and induce tumorigenicity of multiple myeloma cells by targeting PTEN [36]. We found miR-19b-3p was higher in ESCA samples, plasma, and cells than in the healthy group, suggesting that miR-19b-3p may play a tumorigenic role in the progression of ESCA. The current research on miR-19b-3p is limited to the analysis of its expression profile in different ESCA samples [19]. Thus, the present study is the first to explore the migration function of miR-19b-3p transferred by exosomes in ESCA cells.

Exosomes play important roles in cancer progression, it has been reported that exosome levels may be useful as an independent prognostic factor for esophageal squamous cell carcinoma patients [7]. Studies have reported the effect of exosome-derived miRNA on ESCA and esophageal squamous cell carcinoma [10,12,37,38]. For example, miR-21 was highly expression in exosomes in ESCA, and anti-miR-21 inhibited the chemotherapy resistance of ESCA *in vitro* [37]; exosome-derived miR-339-5p mediates radiosensitivity by targeting Cdc25A in locally advanced esophageal squamous cell carcinoma [38]. miR-19b-3p In the present study, an *in vitro* cell co-culture model that simulated ESCA microenvironment was established and the effects of ESCA-derived exosomes transferred miR-19b-3p on EC9706 cells was further explored. We found that miR-19b-3p transferred by exosomes from donor cells could enter the recipient cells and regulate their apoptosis, migration and invasion. It is suggested that miR-19b-3p transferred by exosomes may play a role as an oncogene in ESCA.

miR-19b-3p, which is a complex molecular regulatory network with target genes and related signaling pathways, plays a key role in the development of cancer [39,40]. According to the literature review and TargetScan prediction, we chose tumor-associated gene PTEN for further research. PTEN is a tumor suppressor gene on human chromosome 10 with bispecific phosphatase activity [41]. Mutation or inactivation of PTEN gene is closely related to tumorigenesis, metastasis, deterioration and prognosis [41]. In ESCA, PTEN expression is reduced, which is related to the degree of ESCA tumor differentiation, lymph node metastasis and depth of invasion [42]. Our research showed that miR-19b-3p transferred by exosomes can effectively target the inhibition of PTEN expression in recipient cells, suggesting that PTEN may be one of the pathways for miR-19b-3p transferred by exosomes to promote ESCA progression. PTEN can exit from the cell through exosomal export or secretion and has a tumor suppressor function in adjacent cells [43]. Studies have shown that PTEN is down-regulated in many tumors and integrates a complex signal network system directly or indirectly to suppress tumors [44]. It has been reported that exosome-mediated miR-155 transfer contributes to hepatocellular harcinoma hell proliferation by targeting PTEN [45]. PTEN plays an important regulatory role in the metastasis of tumor cells, astrocyte-derived exosomes mediate an intercellular transfer of PTEN-targeting microRNAs to metastatic tumour cells [46]. Ovarian cancer cell-secreted exosomal miR-205 induces angiogenesis to promote metastasis via the PTEN-AKT pathway [47]. Consistent with previous reports, our data indicated that overexpression of PTEN in the recipient EC9706 cells can promote cell death, inhibit cell migration and invasion, and regulate EMT-related proteins. More importantly, our research suggested that miR-19b-3p may affect the expression of downstream EMT-related proteins through the PTEN pathway to promote the migration of receptor EC9706 cells.

In summary, our study found that miR-19b-3p expression is abnormally high in ESCA. In addition, miR-19b-3p transferred by exosomes can affect the apoptosis and migration of recipient ESCA cells through the PTEN pathway.

## Data Availability

The analyzed data sets generated during the study are available from the corresponding author on reasonable request.

## Abbreviations

AKT: protein kinase B
EMT: epithelial-mesenchymal transition
ESCA: esophageal cancer
PI3K: phosphatidylinositol 3 kinase
PTEN: phosphatase and tension homolog
RT-qPCR: real-time quantitative PCR
